# Gender-Sensitive Depression Scales: A Review of Male-Specific Assessment Tools

**DOI:** 10.3390/diagnostics16060925

**Published:** 2026-03-20

**Authors:** Dominika Jabłonka, Maja Łądkowska, Natalia Kossak, Stefan Modzelewski, Napoleon Waszkiewicz

**Affiliations:** Department of Psychiatry, Medical University of Bialystok, pl. Wołodyjowskiego 2, 15-272 Białystok, Poland; 39748@student.umb.edu.pl (M.Ł.); 39653@student.umb.edu.pl (N.K.); stefan.modzelewski@sd.umb.edu.pl (S.M.); napoleon.waszkiewicz@umb.edu.pl (N.W.)

**Keywords:** gender difference, male depression, suicide, scale

## Abstract

**Background**: Depression in men often goes unrecognized, even though it leads to high rates of suicide. Men may show symptoms that are external, behavioral, or physical, which traditional assessment tools focused on internal symptoms do not adequately reflect. **Methods**: A narrative review was carried out to gather evidence on depression scales tailored for men. We searched PubMed up to November 2025 for studies discussing the development, validation, and clinical use of the Gotland Male Depression Scale (GMDS), the Male Depression Risk Scale (MDRS-22 and MDRS-7), and the Gender-Sensitive Depression Screening scale (GSDS-26). We organized the findings by instrument. **Results**: The studies indicate that male-sensitive scales capture symptom domains such as emotional suppression, anger, risk-taking behaviors, substance misuse, and somatic complaints. The GMDS has demonstrated applicability across psychiatric, somatic, and paternal perinatal populations. The MDRS-22 and MDRS-7 were particularly sensitive to externalizing symptom patterns associated with male presentations of depression and behavioral profiles linked to elevated suicide risk. The GSDS-26 integrates both prototypical and externalizing symptoms, enabling the identification of diverse depressive profiles. However, the current evidence base remains limited due to a reliance on non-clinical samples and the scarcity of long-term and cross-cultural validation studies. **Conclusions**: Male-sensitive depression scales may serve as useful complementary screening tools that improve recognition of male-typical depressive presentations and behavioral patterns associated with increased suicide risk. Further clinical and longitudinal research is needed to confirm their diagnostic accuracy and clinical utility.

## 1. Introduction

According to a World Health Organization (WHO) fact sheet updated on 29 August 2025, about 332 million people around the world suffer from depressive disorders. This accounts for roughly 5.7% of the global adult population. The same source notes that depression is about 1.5 times more common in women than in men [1]. However, WHO global health estimates for 2021 show that men die by suicide at nearly twice the rate of women. The global age-standardized suicide rates are 12.3 per 100,000 in males compared to 5.6 per 100,000 in females [2]. Research consistently shows that while women attempt suicide more frequently, men are much more likely to die from it. A large multi-regional study by Freeman et al. reported notably higher rates of completed suicide among men despite fewer attempts [3]. Clinical data also indicate that male suicide attempts often involve more lethal methods and a lower chance of rescue [4]. Comparative studies confirm that men are more likely to choose highly fatal methods, contributing to their higher suicide mortality [5]. One likely explanation for this difference is that men report fewer classic internalizing symptoms of depression, leading to more undiagnosed cases. Shi et al. showed that men are less likely to acknowledge typical depressive symptoms such as sadness, crying, or guilt. Instead, they are more likely to display irritability, anger, substance use, or risk-taking behaviors [6]. The authors stressed the importance of including male-specific symptoms of depression in diagnostic frameworks to improve detection. Similar findings appear in other studies, suggesting that standard diagnostic tools, which focus mainly on internalizing symptoms, do not fully capture the externalizing and behavioral aspects of male depression [7,8]. In response to these gender-based differences in symptom expression, several assessment tools sensitive to gender have been created. These include the Gotland Male Depression Scale (GMDS) [9,10], initially proposed in Rutz’s concept of “male depression,” the Gender-Sensitive Depression Screening scale (GSDS-26) [11], and the Male Depression Risk Scale (MDRS-22) [7], along with its short form, MDRS-7 [12]. These tools consider factors such as emotional suppression, anger, substance misuse, and risk-taking behaviors. Their goal is to provide a better assessment of depressive symptoms in men. Although male-sensitive instruments demonstrate promising psychometric properties, most validation studies rely on community samples, with limited evidence of systematic implementation in psychiatric settings. The scales reviewed in this article are not designed to diagnose specific depressive disorders such as Major Depressive Disorder, Persistent Depressive Disorder, or bipolar depression. Instead, they assess depressive symptom patterns—particularly externalizing and behavioral manifestations—that may occur across different depressive conditions. The aim of this review is to synthesize current evidence on male-specific depression scales, evaluate their effectiveness compared with traditional instruments (including the Beck Depression Inventory (BDI) and the Hamilton Depression Rating Scale (HAM-D)), and examine the translational gap between research findings and routine psychiatric implementation.

## 2. Materials and Methods

### 2.1. Search Strategy

This narrative review explored the available evidence on assessment tools specifically designed to evaluate depression in men. A PubMed search was performed up to November 2025. The search strategy used the following query: (“Male Depression Risk Scale”[Title/Abstract] OR MDRS[Title/Abstract] OR “Gotland Male Depression Scale”[Title/Abstract] OR GMDS[Title/Abstract] OR “Gender-Sensitive Depression Screening”[Title/Abstract] OR GSDS[Title/Abstract]) AND depression Additional records were identified by screening the reference lists of relevant articles.

### 2.2. Eligibility Criteria

We included peer-reviewed studies that: (1) described the development, validation, or cultural adaptation of MDRS-22, MDRS-7, GMDS, or GSDS/GSDS-26; and/or (2) reported the psychometric properties or clinical useful-ness of these scales. We excluded conference abstracts, non-peer-reviewed reports, and studies focused solely on pediatric populations.

### 2.3. Search Limits and Study Selection

The search was limited to records available in PubMed up to November 2025. No formal start date restriction was applied. Only articles with sufficient information available in English for data extraction and interpretation were considered. The search yielded 125 records. After title and abstract screening, 46 full-text articles were assessed for eligibility. Of these, 14 studies met the inclusion criteria and were included in the final narrative synthesis. Additional relevant publications were identified through manual screening of reference lists.

### 2.4. Data Extraction and Synthesis

From the eligible studies, we extracted data on sample characteristics, scale structure, reliability measures, validity results, and, when available, information on screening performance or suicide-related correlates. Because of substantial heterogeneity in study design, populations, and reported outcomes, findings were synthesized narratively and organized by instrument. Traditional measures such as the Beck Depression Inventory (BDI) and the Hamilton Depression Rating Scale (HAM-D) were included as comparative reference instruments rather than as primary targets of the review.

## 3. Results

### 3.1. Sex Differences in Depressive Symptomatology

Sex differences in depressive symptomatology arise from a combination of biological, psychological, and socio-cultural mechanisms that influence how men and women experience, interpret, and report mood-related distress. These mechanisms shape emotional expression, stress reactivity, and behavioral responses, contributing to observable differences in depressive presentations between the sexes. Sex hormones exert widespread effects on the brain through genomic and non-genomic pathways and influence neural systems involved in mood regulation, cognition, pain perception, and stress responsiveness [13]. Such neurobiological factors provide a plausible background for sex-specific manifestations of depression, potentially contributing to differences in symptom endorsement despite a shared underlying disorder. Fluctuations in sex hormone levels, particularly across the female lifespan, may further amplify somatic sensitivity and emotional reactivity, resulting in symptom profiles that are more readily captured by traditional depression scales [14]. Structural neuroimaging studies have reported sex differences in gray matter volume in regions implicated in emotional regulation and stress processing, including the amygdala, hippocampus, insula, and putamen [15]. While these findings suggest potential sex-specific vulnerability patterns, direct associations between regional brain volume and the expression of depressive symptoms remain insufficiently established. Clinically, men diagnosed with depression more frequently exhibit externalizing features such as alcohol and substance misuse, risk-taking behavior, and impaired impulse control, whereas women tend to report higher levels of core depressive symptoms, including dysphoric mood, appetite or weight changes, and sleep disturbances [16,17]. In addition, women consistently provide more complex and differentiated verbal descriptions of emotional experiences than men, even after controlling for verbal intelligence [18]. Depressed men, by contrast, are more likely to describe distress in terms of bodily sensations and physical processes, whereas women more often use established psychological concepts [19]. Growing evidence indicates that men are therefore more likely to report symptoms that fall outside standard diagnostic criteria, including externalizing and behavioral features, which may contribute to under-detection of depression when traditional internalizing-focused scales are used [20]. Together, these biological and psychological factors provide a coherent framework explaining why depressive symptoms diverge between men and women and why conventional diagnostic instruments may insufficiently capture male-specific depressive presentations. These differences provide the conceptual rationale for the development of male-sensitive assessment tools, which attempt to capture externalizing, behavioral, and somatic manifestations of depression that may be underrepresented in traditional measures.

### 3.2. Beck Depression Inventory (BDI)

Developed in 1961 by Aaron T. Beck, the BDI is a 21-item self-report questionnaire designed to assess the severity of depressive symptoms in both psychiatric and non-psychiatric populations. The revised version, BDI-II, is currently the most widely used form and has been extensively validated, demonstrating strong convergent, discriminant, concurrent, and factorial validity [21]. The BDI-II shows high reliability and robust correlations with measures of depression and anxiety, and effectively differentiates individuals with and without depression in both primary care and hospital settings [22]. Importantly, the BDI is a symptom severity measure and does not constitute a diagnostic instrument. Across multiple studies, women tend to obtain slightly higher mean BDI-II scores than men, particularly on items assessing crying, appetite and weight changes, and loss of sexual interest. These differences may reflect biological, psychological, and socio-cultural factors influencing emotional expression and symptom reporting. In contrast, men with depression more frequently report alcohol and substance misuse or other externalising behaviours, domains that are not directly assessed by the BDI [20,23]. Importantly, evidence from clinical samples indicates that the BDI may be less effective in detecting depression in men. In a study of elderly psychiatric patients with major unipolar depression, the BDI failed to identify clinically diagnosed depression in approximately one quarter to one half of male patients, depending on the cut-off applied [24]. Together, these findings suggest that while the BDI-II is a reliable and valid measure of depressive symptom severity, its focus on internalising symptoms may limit sensitivity to externalising and behavioural manifestations of depression more commonly reported by men. This limitation may contribute to systematic underestimation of depressive severity in male populations and highlights the need for complementary gender-sensitive assessment tools.

### 3.3. Hamilton Depression Rating Scale (HAM-D)

The HAM-D was developed in the 1950s as a clinician-rated instrument for assessing the severity of depressive symptoms. Each item is scored using anchor points reflecting increasing symptom intensity, with clinicians considering both severity and frequency when assigning ratings. Owing to its structured format and long-standing use, the HAM-D remains one of the most widely applied instruments in both clinical practice and depression research [25,26]. As the scale was originally designed for hospitalized patients, it places particular emphasis on melancholic features and somatic manifestations of depression. Consistent with this design, several studies have shown that overall HAM-D scores are often similar between men and women despite clear sex differences in symptom profiles. In a large sample of 930 hospitalized and outpatient patients with major depression, no sex differences were observed in median total HAM-D scores or the number of endorsed symptoms, even though symptom patterns differed between men and women [27]. Comparable findings were reported in a study using the 17-item HAM-D (HDRS-17), in which mean total scores did not differ significantly between male and female patients. However, women more frequently endorsed somatic symptoms assessed by the scale, whereas men were less likely to report such complaints [14]. This pattern aligns with broader evidence that women tend to report more somatic and internalising symptoms, while men may underreport these features. Psychometric evaluations indicate that although the HAM-D demonstrates acceptable internal consistency, several items show limited sensitivity to changes in depressive severity and suboptimal interrater or retest reliability. Moreover, content validity is constrained by the scale’s narrow focus on melancholic and somatic domains, while externalising or behavioural symptoms are largely absent. Factor-analytic studies have identified a multidimensional structure with limited replicability across samples [28]. Cross-cultural studies further suggest that interpretation of HAM-D scores may be influenced by cultural norms regarding symptom expression. Validation of translated versions, such as the Chinese HAM-D, has demonstrated good reliability but also revealed variability in factor structure, underscoring the importance of contextual factors in symptom reporting [29]. Taken together, the HAM-D provides a reliable clinician-rated measure of depressive severity, particularly for melancholic and somatic symptoms. However, its limited coverage of externalising and behavioural features, combined with evidence of sex differences in symptom endorsement, suggests that the scale may underestimate depressive severity in men-especially in those whose distress is expressed outside traditional internalising domains.

### 3.4. Male Depression Risk Scale (MDRS-22)

MDRS-22 was developed in 2013 to assess depressive symptomatology that may be particularly salient in men and insufficiently captured by traditional depression scales. The scale was constructed using online, non-clinical community samples, beginning with a broad pool of items derived from existing literature and clinical observations of male-typical depressive presentations. Two empirical studies informed scale development: an initial exploratory factor analysis conducted in a male-only sample to refine items, followed by a mixed-sex sample to confirm the factor structure and examine sex differences. Subsequent validation studies have provided additional support for the psychometric properties and clinical relevance of the MDRS-22. A longitudinal study by Rice et al. employed multidomain latent growth modelling across three measurement waves (baseline, 3 months, 6 months) to compare symptom trajectories assessed by the MDRS-22 and the Patient Health Questionnaire-9 (PHQ-9) in men reporting mental health difficulties The MDRS-22 demonstrated good internal consistency across time (Cronbach’s α and McDonald’s ω) and comparable reliability to the PHQ-9. Importantly, baseline MDRS-22 scores, but not PHQ-9 scores, significantly differentiated men who were engaged in mental health treatment from those who were not, indicating greater sensitivity of the MDRS-22 to clinically relevant group differences. Trajectory analyses further showed that higher baseline MDRS-22 scores were associated with a slower rate of symptom improvement over six months, whereas baseline PHQ-9 severity was not related to change over time. Despite strong longitudinal correlations between MDRS-22 and PHQ-9 scores, these findings suggest that the MDRS-22 captures distinct symptom domains that are both clinically meaningful and prognostically relevant for male-typical depressive presentations. The MDRS-22 is a self-report instrument comprising six domains associated with externalising or male-salient depressive symptoms: emotional suppression, drug use, alcohol use, anger and aggression, somatic symptoms, and risk-taking behaviours. Items are rated on an 8-point Likert scale (0–7), with higher scores indicating greater symptom endorsement. Sub-scale scores can be summed to yield a total score reflecting the overall burden of externalising depression risk. In the original validation study, higher MDRS-22 scores were associated with greater conformity to traditional masculine norms, supporting the intended construct validity of the scale. A longitudinal study by Rice et al. demonstrated good internal consistency (α and ω) and sensitivity to symptom change over time among men experiencing mental health difficulties. Notably, the MDRS-22 was more sensitive than the PHQ-9 to changes in externalising symptoms, and higher baseline scores were associated with slower symptom improvement, suggesting potential clinical relevance for monitoring male-typical depressive trajectories [7,30]. Cross-cultural validation has further supported the utility of the MDRS-22. In a large German online sample (n = 1605), the adapted version demonstrated good reliability and validity, with full replication of the original six-factor structure. Higher MDRS-22 scores were associated with greater psychological distress as well as self-reported use of psychotherapy and psychopharmacological treatment. Importantly, the MDRS-22 showed detection performance comparable to that of the PHQ-9 in identifying men reporting psychological distress, diagnosed mental disorders, or treatment engagement. The authors concluded that the MDRS-22 represents a valid cross-culturally applicable screening instrument for identifying male-typical depressive and distress-related symptom patterns [31]. More recent work has examined measurement invariance across age groups. A study comparing younger (18–64 years) and older (65+ years) men found that full age invariance was not supported, indicating that the expression and measurement of male-typical depressive symptoms may vary across the lifespan [32]. Overall, available evidence supports the MDRS-22 as a psychometrically sound instrument for capturing externalising dimensions of depression in men that are underrepresented in conventional scales. However, findings also highlight the importance of considering demographic factors, particularly age, when interpreting scores.

### 3.5. Male Depression Risk Scale-7 (MDRS-7)

MDRS-7 was developed as a brief version of the MDRS-22 to facilitate the assessment of male-typical depressive symptoms in time-limited clinical settings, such as primary care. While the full MDRS-22 captures a broad range of externalising symptom domains, its length may limit feasibility in routine practice. The MDRS-7 therefore condenses the original instrument into seven items while retaining its core conceptual domains. The first formal validation of the MDRS-7 was conducted in a large community-based sample of Australian men (n = 1605), including both younger (18–64 years) and older (65+ years) age groups. Item reduction procedures and factor analyses demonstrated that the MDRS-7 captured a single underlying construct corresponding to the male-typical depression phenotype, with a near-perfect correlation with the full MDRS-22 (r = 0.94). The scale showed acceptable internal consistency across age groups and time points [12]. Importantly, the MDRS-7 demonstrated clinical utility beyond traditional depression measures. When used in combination with the PHQ-9, the scale differentiated men with externalising, prototypic, mixed, and minimal depressive symptom profiles. Men presenting with mixed symptomatology (elevated MDRS-7 and PHQ-9 scores) exhibited the highest levels of psychological distress and current suicidality, while men with predominantly externalising symptoms also showed significantly elevated suicide risk despite subthreshold PHQ-9 scores. Furthermore, moderate MDRS-7 symptom severity at baseline predicted depressive symptoms at follow-up, independent of prior depression diagnosis, suggesting potential prodromal value. Collectively, these findings support the MDRS-7 as a brief, psychometrically acceptable screening tool for identifying male-typical depressive and suicide-related risk profiles that may be overlooked by prototypic depression scales. Additional evidence for the clinical relevance of the MDRS-7 comes from a study of 606 Australian men, which examined the role of male-typical depressive symptoms in mediating the relationship between avoidant coping and suicidal or self-harm ideation. The MDRS-7 demonstrated satisfactory internal consistency (Cronbach’s α = 0.778), and higher MDRS-7 scores were associated with greater suicidal/self-harm ideation, as assessed using item 9 of the PHQ-9 [33]. Overall, available cross-sectional validation studies support the MDRS-7 as a useful brief screening instrument for male-typical depressive symptoms. However, empirical evidence remains limited, and most studies have relied on non-clinical samples. Further research in clinical and more diverse populations is needed to establish optimal cut-off scores, diagnostic accuracy, and longitudinal utility.

### 3.6. Gotland Male Depression Scale (GMDS)

GMDS was developed by Wolfgang Rutz to capture depressive symptomatology that may be under-recognized in men when assessed using traditional internalising-focused instruments. The scale was grounded in the conceptual framework of “male depression,” which emphasizes atypical and externalising manifestations such as irritability, aggression, impulsivity, substance misuse, stress intolerance, and difficulties with emotional regulation [9,10]. These features were proposed to contribute to misdiagnosis and underdetection of depression in men, particularly in clinical settings relying on conventional diagnostic criteria. The GMDS is a 13-item self-report instrument assessing depressive symptoms experienced over the preceding month. Items are rated on a four-point Likert scale (0–3), resulting in a total score that reflects symptom severity. The scale comprises two subdomains—distress and depression—and complements classical depressive symptoms (e.g., low mood, sleep disturbance) with male-salient behavioural and emotional features. Evidence supporting the clinical utility of the GMDS includes studies that directly compare its screening performance with that of standard depression instruments. In a clinical sample of 233 psychiatric outpatients, the GMDS demonstrated higher sensitivity and overall screening accuracy for detecting depressive symptoms in men compared with the PHQ-9, whereas the PHQ-9 showed superior screening performance in women. These findings suggest that the GMDS may be particularly suitable for screening depression in male populations. The relevance of the GMDS for identifying suicide-related risk in men has also been supported by empirical evidence. In a large study involving nearly 600 men drawn from clinical, somatic, and non-clinical populations, higher GMDS scores were associated with greater severity of depressive symptoms and increased suicidal behaviour. The authors emphasized that both risk and protective factors for depression and suicidality in men are multidimensional, encompassing psychological, socioeconomic, familial, and environmental domains [34]. The GMDS has been further applied in specific male populations in which depressive symptoms may be particularly difficult to detect. Studies of paternal postpartum depression have shown that the GMDS identifies a higher proportion of fathers at risk compared with conventional instruments such as the Edinburgh Postnatal Depression Scale, underscoring the potential under-recognition of male-typical depressive symptoms when gender-neutral screening tools are used. At the same time, these findings highlight the need for culturally adapted and context-specific assessment instruments tailored to fathers in diverse sociocultural settings [35]. Cross-cultural validation studies have provided additional support for the psychometric robustness of the GMDS. The Chinese version of the scale demonstrated good reliability and a predominantly unidimensional structure, supporting its applicability in Asian populations. Nonetheless, variability in certain items, particularly those related to substance misuse, suggests that cultural context may influence the expression and measurement of male-typical depressive symptoms [36]. The GMDS has also demonstrated clinical relevance in men with serious somatic illnesses, such as prostate cancer. Factor-analytic studies in oncology populations have identified a stable two-factor structure distinguishing changes in emotional and somatic functioning from depressed mood, reinforcing the notion that male depression in the context of chronic illness may present as a complex and multifaceted construct that is not fully captured by conventional diagnostic models [37]. Overall, the GMDS represents the first explicitly gender-sensitive depression screening instrument developed specifically to address male-typical depressive symptomatology. It constitutes a clinically meaningful tool for detecting depressive symptoms in men, particularly in contexts where externalising, behavioural, or somatic features predominate. While it does not replace standard diagnostic instruments, available evidence suggests that the GMDS may serve as a valuable complementary measure to improve the identification of depression and suicide-related risk in male populations.

### 3.7. Gender-Sensitive Depression Screening (GSDS)

GSDS was originally developed to assess both prototypical and externalising depressive symptoms that may be insufficiently captured by conventional depression measures. The initial version of the instrument comprised 33 items reflecting seven theoretically derived domains identified in contemporary research on male depression: depressiveness, stress, emotional regulation or suppression, aggression, risk-taking behaviour, hyperactivity, and alcohol use. Following psychometric evaluation and item reduction procedures, the scale was refined to a final 26-item version, the GSDS-26. In its final form, the GSDS-26 comprises six subscales assessing emotional control, risky behaviour, stress perception, depressiveness, aggressiveness, and alcohol consumption. Validation of the GSDS-26 was conducted in a large non-clinical sample recruited between 2021 and 2022, including 1087 adults (746 men and 341 women). The scale was designed as a gender-sensitive screening instrument applicable to both sexes, while explicitly incorporating symptom dimensions shown to be particularly salient in men. For this reason, although the GSDS-26 is not strictly a male-specific instrument, it was included in the present review as a gender-sensitive tool that captures male-salient depressive symptom patterns. Psychometric evaluation supported the multidimensional structure of the GSDS-26, with confirmatory analyses confirming the six-factor model. Convergent validity was examined using established instruments, including the Beck Depression Inventory (BDI), the Gotland Male Depression Scale (GMDS), and the Conformity to Masculine Norms Inventory (CMNI-22). The GSDS-26 showed moderate to strong correlations with measures of depressive symptom severity and masculine norm conformity, supporting its construct validity and theoretical grounding in gender-sensitive models of depression [11]. The GSDS-26 demonstrated high overall internal consistency (Cronbach’s α = 0.92), with strong reliability across most subscales (α = 0.80–0.85), although the alcohol consumption subscale showed lower reliability (α = 0.63). Construct validity was supported by strong correlations with the BDI (r = 0.76) and the GMDS (r = 0.72). Within this sample, men scored significantly higher than women on the GSDS-26 total score and on subscales assessing emotional control, risky behaviour, and alcohol consumption, suggesting enhanced sensitivity to male-salient depressive features compared with traditional internalising-focused measures. Additional clinical evidence for the relevance of the GSDS comes from a German forensic psychiatric study comparing GSDS and BDI-II scores in matched male and female patients. Among men, GSDS scores showed significant associations with internalising depressive symptoms and were higher in those with a history of suicide attempts, whereas no such associations were observed in women. In contrast, female patients with a suicidal history exhibited higher scores on the BDI-II. These findings suggest that externalising behaviours may function as markers of depressive pathology in men but not necessarily in women, highlighting the gender-specific interpretability of GSDS scores [38]. Taken together, available evidence indicates that the GSDS-26 is a psychometrically sound, gender-sensitive screening instrument that captures both internalising and externalising dimensions of depressive symptomatology. However, most validation studies have been conducted in non-clinical or highly specific populations, and evidence from large, diverse clinical samples remains limited. Consequently, while the GSDS-26 appears to be a valuable complementary tool—particularly for identifying male-typical depressive presentations—it should not be considered a standalone diagnostic instrument, and further research is required to establish its clinical accuracy and generalisability.

## 4. Discussion

Table 1 summarizes the coverage of key symptom domains across male-sensitive (GMDS, MDRS-22, MDRS-7, GSDS) and conventional (BDI, HAM-D) depression scales. The comparison highlights the broader representation of externalizing symptoms in male-specific instruments, whereas traditional scales predominantly focus on prototypical internal depressive symptoms. A more detailed comparison of the psychometric characteristics, strengths, limitations, and clinical relevance of these instruments is provided in Table S1 in the Supplementary Material. In addition, Supplementary Table S2 presents a structured summary of the key studies evaluating the reviewed instruments, including sample characteristics, scales used, and main findings.

This narrative review synthesises current evidence on male-sensitive depression scales and highlights important conceptual and psychometric differences among instruments designed to capture depressive symptomatology in men. As summarised in Table 1, the reviewed scales—GMDS, MDRS-22, MDRS-7, and GSDS-26—share a common aim of addressing depressive presentations that traditional internalising frameworks may insufficiently identify, yet differ substantially in scope, structure, and intended clinical application. The Gotland Male Depression Scale represents the earliest gender-sensitive approach to depression assessment and has demonstrated clinical utility in identifying depressive presentations associated with elevated suicide risk in men across psychiatric, somatic, and community settings. The MDRS-22 extends this framework by focusing explicitly on externalising symptom trajectories and male-salient risk patterns, offering greater sensitivity to symptom change over time and potential prognostic value. Its short form, the MDRS-7, prioritises feasibility in time-limited clinical contexts and appears particularly useful for screening male-typical depressive and suicide-related risk profiles in primary care. In contrast, the GSDS-26 adopts a broader gender-sensitive model that integrates both prototypical and externalising depressive symptoms, enabling the identification of heterogeneous symptom profiles while remaining applicable to both men and women. An important observation emerging from this comparison is that none of the reviewed male-sensitive scales (GMDS, MDRS-22, MDRS-7, GSDS-26) directly assesses suicidality through explicit items addressing suicidal ideation or intent. In contrast, traditional instruments such as the BDI and HAM-D include dedicated suicide-related items. Conversely, these conventional scales do not systematically operationalise domains such as risk-taking behaviour or substance misuse, and they only partially capture irritability, with BDI including it more consistently than HAM-D. This divergence highlights a conceptual gap: while male-sensitive scales focus on behavioural and externalising risk markers potentially associated with suicide vulnerability, traditional tools retain direct assessment of suicidal thoughts but may overlook behavioural patterns that precede or mask suicidal crises in men. This asymmetry may partially contribute to the paradox of high suicide mortality among men despite comparable or lower detected rates of depressive disorders. Despite these advances, the empirical base supporting male-sensitive depression scales remains limited in both scope and volume. The number of independent validation studies remains relatively small, and most available data derive from cross-sectional designs conducted in non-clinical or highly specific populations. Moreover, externalising symptoms assessed by these instruments are not specific to depression and may overlap with substance use disorders, personality traits, or socioculturally shaped expressions of masculinity, necessitating cautious interpretation of elevated scores. Elevated scores should be interpreted cautiously and in conjunction with broader clinical assessment. Taken together, the available evidence supports the use of male-sensitive depression scales as complementary screening tools that capture distinct but overlapping dimensions of depressive symptomatology in men. Rather than competing instruments, GMDS, MDRS, and GSDS address different aspects of male depression and may be optimally applied in combination or selected according to clinical context. In addition to the limitations of the currently available evidence, several limitations of the present review should also be acknowledged. First, the literature search was limited to the PubMed database, which may have resulted in the omission of relevant studies indexed elsewhere. Second, as this review followed a narrative rather than systematic approach, the study selection and synthesis process may be more susceptible to selection bias. Finally, the relatively small number of available validation studies limited the possibility of conducting a more structured comparison of psychometric properties across instruments. Future research should prioritise large-scale clinical validation, cross-cultural studies, and prospective designs to determine whether the use of male-sensitive assessment strategies improves diagnostic precision, treatment outcomes, and suicide prevention efforts among men.

## Figures and Tables

**Table 1:** Symptom Domain Coverage in Male-Sensitive and Traditional Depression Assessment Tools (“+” indicates that the domain is explicitly assessed within the scale structure; “−” indicates absence of a specific domain).

Scales	GMDS	MDRS-22	MDRS-7	GSDS	BDI	HAM-D
Items	13	22	7	26	21	17
Externalizing symptoms	+	+	+	+	−	−
Anger/irritability	+	+	+	+	+	−
Emotional suppression	−	+	+	+	−	−
Sleep problems	+	−	−	−	+	+
Risk-taking	−	+	+	+	−	−
Substance misuse	+	+	+	+	−	−
Overwork	+	−	−	−	−	−
Somatic complaints	+	+	+	+	+	+
Suicidality	−	−	−	−	+	+
Prototypical internal symptoms	+	−	−	+	+	+

## Data Availability

No new data were created or analyzed in this study. Data sharing is not applicable to this article.

## Supplementary Materials

The following supporting information can be downloaded at: https://www.mdpi.com/article/10.3390/diagnostics16060925/s1, Table S1: Extended Comparison of Male-Sensitive Depression Assessment Tools. Table S2: Summary of key studies evaluating male-sensitive depression assessment scales.
