# Supplementary material for: Gender-Sensitive Depression Scales: A Review of Male-Specific Assessment Tools
Source: Diagnostics (Basel). 2026 Mar 20;16(6):925. doi: 10.3390/diagnostics16060925 (PMC13024911; doi:10.3390/diagnostics16060925)
Supplement: Supplementary file 1 [file diagnostics-16-00925-s001.zip › Table S1.pdf]

Table S1. Extended Comparison of Male-Sensitive Depression Assessment Tools.

Scales	Core symptom domains	Externalising symptoms	Administration	Main strengths	Reliability (Cronbach's α)	Suicide-related relevance	Key limitations
GMDS	Distress; depressed mood; emotional dysregulation	Irritability; aggression; impulsivity; substance misuse; stress intolerance; somatic complaints	Self-report; 4-point Likert (0–3); past month	First gender-sensitive depression scale developed specifically for men; improves detection of male-typical depressive presentations; higher screening sensitivity in men than PHQ-9 in clinical samples; applicable in medical and non-psychiatric settings	High internal consistency reported across studies ($\alpha \approx$ 0.90–0.93)	Higher GMDS scores associated with suicidal behaviour and suicide-related risk; useful for identifying at-risk men in clinical and somatic populations	Fixed symptom profile; variable factor structure across studies; limited longitudinal validation; cultural variability in substance-related items; not a diagnostic instrument
MDRS-22	Emotional suppression; anger/aggression; somatic symptoms	Alcohol use; drug use; risk-taking; anger/aggression	Self-report; 8-point Likert (0–7); past month	Captures male-salient externalising symptom patterns; multidimensional structure; sensitivity to change over time; differentiates treatment-seeking vs non-treatment men; prognostic value for symptom trajectories	α = 0.842 (baseline) α = 0.879 (3 months) α = 0.871 (6 months); Good internal consistency across studies (α and ω reported as satisfactory to good); comparable to PHQ-9	Indirect: associated with psychological distress and treatment uptake; identifies symptom patterns linked to elevated suicide risk	Non-clinical samples; not a diagnostic scale; age-related measurement non-invariance; interpretation may vary across lifespan

MDRS-7	Emotional suppression; anger/aggression; somatic symptoms	Alcohol use; drug use; risk-taking; anger/aggression	Self-report; Likert-type scale; brief administration suitable for primary care	Very brief and feasible; retains core domains of MDRS-22; near-perfect correlation with full scale (r = 0.94); identifies male-typical depressive profiles; adds information beyond PHQ-9	Acceptable internal consistency ($\alpha \approx$ 0.78); comparable reliability across age groups	Associated with suicidal/self-harm ideation; identifies elevated suicide risk even with subthreshold PHQ-9 scores; mediates relationship between avoidant coping and suicidality	Non-clinical samples; limited number of validation studies; lack of established clinical cut-offs; limited longitudinal and clinical validation
GSDS	Depressiveness; stress perception; emotional control; aggressiveness; risky behaviour; alcohol consumption	Aggressiveness; risky behaviour; alcohol use; emotional suppression	Self-report; 4-point Likert scale (0–3); recent weeks	Integrates prototypical and externalising depressive symptoms; multidimensional six-factor structure; higher sensitivity for identifying men at risk than standard internalising-focused tools; captures gender-specific symptom profiles	Total $\alpha \approx$ 0.92; subscales $\alpha \approx$ 0.80–0.85 (alcohol $\alpha \approx$ 0.63)	Scores associated with depressive severity and history of suicide attempts	Predominantly validated in non-clinical or specific populations; limited large-scale clinical validation; lower reliability of alcohol subscale; screening tool, not diagnostic
