# Supplementary material for: Gender-Sensitive Depression Scales: A Review of Male-Specific Assessment Tools
Source: Diagnostics (Basel). 2026 Mar 20;16(6):925. doi: 10.3390/diagnostics16060925 (PMC13024911; doi:10.3390/diagnostics16060925)
Supplement: Supplementary file 1 [file diagnostics-16-00925-s001.zip › Table S2.pdf]

Table S2. Summary of key studies evaluating male-sensitive depression assessment scales.

Study	Country/ sample	Population	Scale evaluated	Comparator scale	Study design	Main psychometric findings	Key clinical implications
Rice et al., 2013	Australia, community sample	Adult men (n=1176)	MDRS-22	None	Scale development and validation study	Adequate internal consistency; Factor structure was confirmed by factor analysis; Good convergent validity with depression severity	SIS scores were associated with increased risk of later suicide; SIS showed the strongest predictive value for possible future suicides;
Rice et al., 2020	Australia,community sample	Adult men (n= 234)	MDRS-22	PHQ-9	Longitudinal study	MDRS demonstrated good reliability; Latent growth moddeling showed good model fit; MDRS was sensitive to longitudinal change and	MDRS was useful adjunctive screening tool for identifying sucicide risk in men

						treatment effects, unlike PHQ-9;	
Walther et al., 2021	Germany, online community sample	Adults (n = 1605; 671 men)	MDRS-22	PHQ-9	Cross-sectional validation study	Six-factor structure replicated; good reliability and validity; detection performance comparable to PHQ-9	Valid screening tool for identifying male-typical depressive symptoms and psychological distress
Owsiany & Fiske, 2024	USA, community sample	Adult men (n = 469; younger 18–64, older 65+)	MDRS-22	None	Cross-sectional psychometric study	Measurement invariance across age groups was not supported	Measurement invariance across age groups was not supported
Herreen et al., 2022	Australia, community sample	Adult men (n = 949)	MDRS-7	MDRS-22, PHQ-9, K10	Cross-sectional study	Seven-item version retained domains of MDRS-22; associated with psychological distress and suicidality; predicted depressive	MDRS-7 may serve as a brief screening tool for male-typical depressive symptoms in primary care

						symptoms at follow-up	
O'Gorman et al., 2022	Australia, community sample	Adult men (n = 606)	MDRS-7	None	Cross-sectional mediation study	Male-type depression symptoms mediated the relationship between avoidant coping and suicidal/self-harm ideation	Screening for male-typical depressive symptoms may help identify men at risk of suicidal ideation
Moon et al., 2025	South Korea, psychiatric outpatient sample	Adult psychiatric outpatients (n = 233)	GMDS	PHQ-9	Cross-sectional validation study	High internal consistency (α = 0.92); strong correlation with PHQ-9 (r = 0.83); comparable ROC performance	GMDS may improve screening for depression in men compared with conventional scales
Chua et al., 2025	Singapore, hospital-based sample	Fathers after childbirth (n = 200)	GMDS	EPDS	Prospective cohort study	GMDS identified a higher proportion of fathers with depressive symptoms compared with EPDS	Male-sensitive scales may improve detection of paternal postpartum depression

Chu et al., 2014	China, men's health clinic	China, men's health clinic	GMDS	BDI-II, AMS	Cross-sectional validation study	High internal consistency (α = 0.933); strong correlation with BDI-II (r = 0.835); acceptable factor structure	Supports use of GMDS for detecting male-typical depressive symptoms in Chinese-speaking populations
Sharpley et al., 2017	Australia and United Kingdom, oncology sample	Men with prostate cancer (n = 329)	GMDS	None	Cross-sectional factor-analytic study	Two-factor structure identified in both samples, reflecting emotional/somatic changes and depressed mood	GMDS may detect male-typical depressive symptoms in men with serious somatic illness
Chodkiewicz et al., 2025	Poland, non-clinical community sample	Adults (n = 1087; 746 men, 341 women)	GSDS	BDI, GMDS, CMNI-22	Cross-sectional validation study	Six-factor structure confirmed (EFA/CFA); high internal consistency (α = 0.92); acceptable reliability across subscales	Supports use of GSDS as a gender-sensitive instrument for assessing male-salient depressive symptoms

Streb et al., 2021	Germany, forensic psychiatric sample	Forensic psychiatric patients (n = 182; matched sample 21 men, 21 women)	GSDS	BDI-II	Cross-sectional clinical study	Externalizing and internalizing depressive symptoms were correlated in men but not in women; higher GSDS scores observed in men with a history of suicide attempts	GSDS may improve identification of male-typical depressive symptoms and suicide risk in forensic populations
